# Posterolateral tibial plateau impaction fractures in patients undergoing primary anterior cruciate ligament reconstruction—An MRI analysis of 556 cases

**DOI:** 10.1002/jeo2.70284

**Published:** 2025-05-26

**Authors:** Andreas Fuchs, Spartak Ovsepyan, Andreas Frodl, Tayfun Yilmaz, Markus Siegel, Matthias Krause, Thomas Stein, Hagen Schmal, Kaywan Izadpanah

**Affiliations:** ^1^ Department of Orthopedic Surgery and Traumatology, Freiburg University Hospital Albert Ludwigs University Freiburg Freiburg Germany; ^2^ Department of Trauma and Orthopaedic Surgery University Medical Center Hamburg‐Eppendorf Hamburg Germany; ^3^ SPORTHOLOGICUM Frankfurt, Center for Sport & Joint Injuries Frankfurt am Main Germany; ^4^ Department of Sports Medicine, Frankfurt am Main Goethe University Frankfurt Frankfurt Germany; ^5^ Department of Orthopedic Surgery University Hospital Odense Odense Denmark

**Keywords:** ACL lesion, concomitant injuries in ACL‐deficient knees, meniscal tear, posterolateral tibial plateau impaction fracture

## Abstract

**Purpose:**

Posterolateral impaction fractures of the tibial plateau are known to be associated with anterior cruciate ligament (ACL) tears. These fractures are often related to high energy pivoting injuries, which is why the frequency of such injuries is of key concern for patients undergoing primary ACL reconstruction. The objective of this study is to evaluate the occurrence of posterolateral tibial fractures, as well as concomitant injuries in patients undergoing primary ACL reconstruction.

**Methods:**

A retrospective case series was conducted to study the occurrence and type of posterolateral tibial impaction fractures in patients undergoing ACL reconstruction between October 2015 and October 2020. Patients records were reviewed to collect patient demographics, exact injury patterns and details about concomitant injuries. Descriptive statistics were performed to determine the incidence of each type of posterolateral tibial plateau impaction fracture, as well as concomitant injuries.

**Results:**

Of the 556 knees with primary ACL reconstruction, a total of 171 posterolateral tibial plateau impaction fractures were identified. 385 patients showed no fracture. Intraoperative arthroscopic examination showed lateral meniscus (LM) tears in 144 cases and medial meniscus (MM) tears in 163 cases. LM posterior root tears were found in 21 patients, MM posterior root tears in eight patients. Medial meniscal ramp lesions were found in a total of 39 knees.

**Conclusion:**

30.8% of the patients showed posterolateral tibial plateau impression fractures, here LM tears were more frequent with the highest incidence in IIIB fractures. MM tears are more frequent in patients without posterolateral impaction fractures, while LM posterior root tears are more frequent than MM posterior root tears among the whole study population. The clinical relevance of this study lies in the exact analysis of posterolateral tibial plateau fractures in patients with ACL lesions, with the resulting therapeutic consequences dependent on the fracture type and concomitant injuries.

**Level of Evidence:** Level III.

**Abbreviations**
ACL: anterior cruciate ligament
LM: lateral meniscus
MM: medial meniscus
MRI: magnetic resonance imaging

## INTRODUCTION

Posterolateral impaction fractures of the tibial plateau are known to be associated with anterior cruciate ligament (ACL) tears [11, 12, 17, 22, 25, 26]. These injuries have been found to occur across a wide spectrum, ranging from bone bruising to impaction fractures with the displacement of cortical or subchondral bone [30]. Bone bruising of the lateral tibial plateau occurs frequently in association with ACL tears. Here, incidences between 58% and 82% have been reported [6, 7, 8, 10, 19, 20, 21, 28, 29]. The occurrence of impaction injuries of the tibial plateau is thought to be associated with higher energy pivoting injuries, as full‐ or high‐grade partial ACL tears are more frequently associated with bone bruising than low‐grade partial tears [29].

A specific classification for posterolateral tibial plateau impaction fractures in patients with ACL tears has been described by Bernholt et al. [2]. By differentiating between subtypes, high‐grade posterolateral tibial plateau impaction fractures are associated with decreased postoperative outcomes after ACL repair and patients with large depression‐type posterolateral tibial plateau impaction fractures (type IIIB) have an increased pivot‐shift laxity on clinical examination [3]. A frequently discussed pathomechanism which underlies these findings is the loss of contact between the lateral meniscus and the tibial plateau in knees with posterolateral fractures, which might have a similar effect to an increased posterior slope [15, 16].

Due to the correlation between ACL‐tears and posterolateral tibial plateau injuries on the one hand and between posterolateral tibial plateau injuries and high energy pivoting injuries on the other hand, the frequency of such injuries in patients undergoing primary ACL reconstruction is of key concern. The objective of this study is therefore to evaluate the occurrence of posterolateral tibial fractures as well as concomitant injuries in patients suffering an ACL lesion.

## MATERIALS AND METHODS

### Study design and patient selection

A retrospective case series was conducted to study the occurrence of posterolateral tibial impaction fractures in patients undergoing ACL reconstruction at our institution. Patients with multiligament injuries of the knee were excluded. The study was approved by the institutional review board of the University Hospital Freiburg and performed in accordance with the Declaration of Helsinki.

A chart review was performed using our electronic medical record system to identify all patients who underwent primary ACL reconstruction between October 2015 and October 2020. Preoperative MRI scans were available for all of these patients. For further patient selection, only patients undergoing primary ACL reconstruction without concomitant cartilage repair or osteotomy procedures were included.

All the patients' records were reviewed in order to collect patient demographics. Additionally, data from operative reports was recorded for each patient, detecting exact injury patterns and details about concomitant injuries, such as meniscal lesions. The diagnosis of meniscal tears in this study required arthroscopic confirmation.

**Figure 1:**
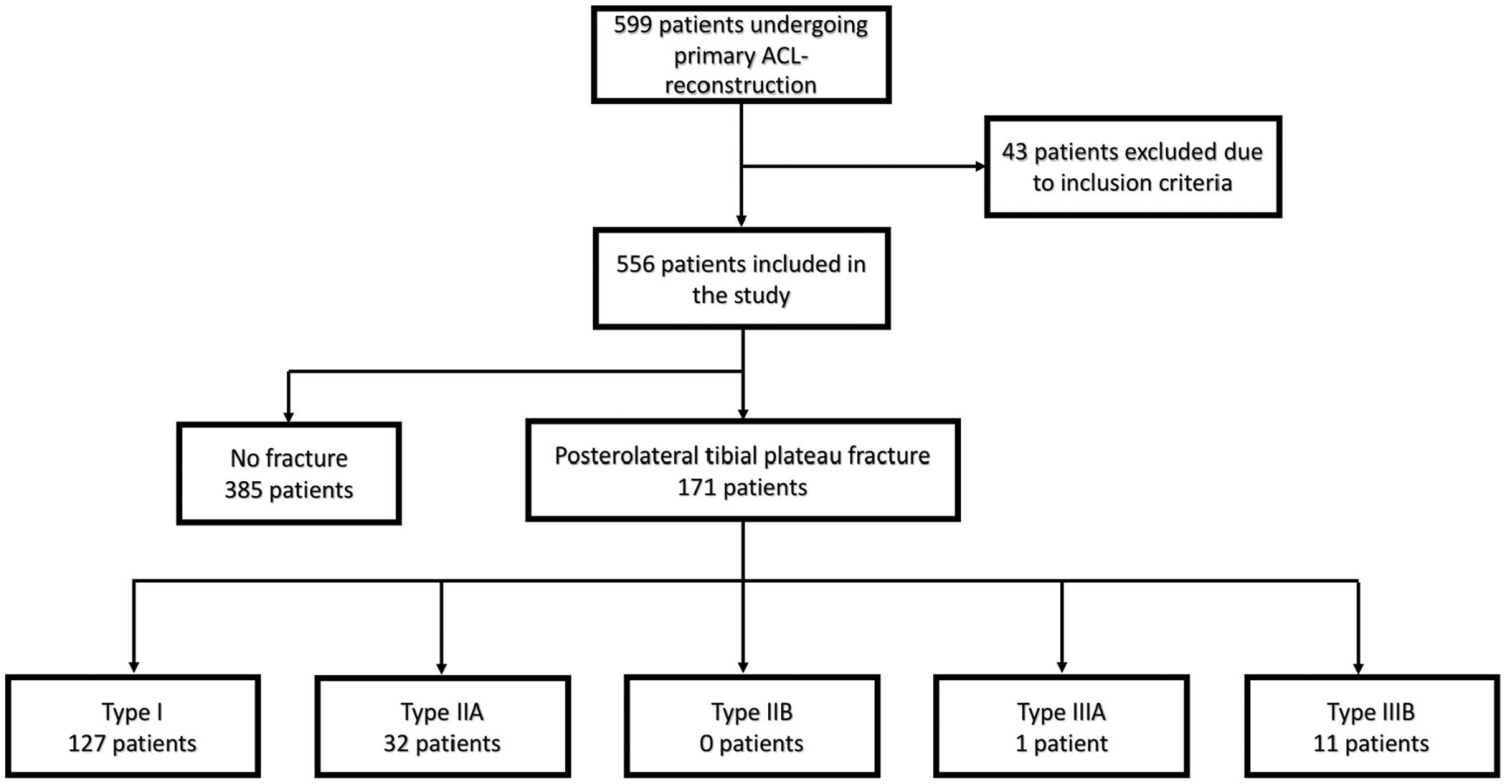
Flowchart of patients who meet the inclusion criteria, the number of patients with posterolateral tibial impaction fractures, and each specific fracture type. ACL, anterior cruciate ligament.

Preoperative MRI scans were reviewed independently by two orthopaedic surgeons for the presence of tibial or femoral oedema, as well as posterolateral tibial plateau impaction fractures. MRI signal change at the posterolateral tibial plateau was only classified as an impaction fracture if the displacement of subchondral or cortical bone at the posterolateral tibial plateau rim was visible. Detected posterolateral tibial plateau fractures were then classified (following the consensus of both investigators after independent classification) according to Bernholt et al. [2] (Figure 1).

**Figure 2:**
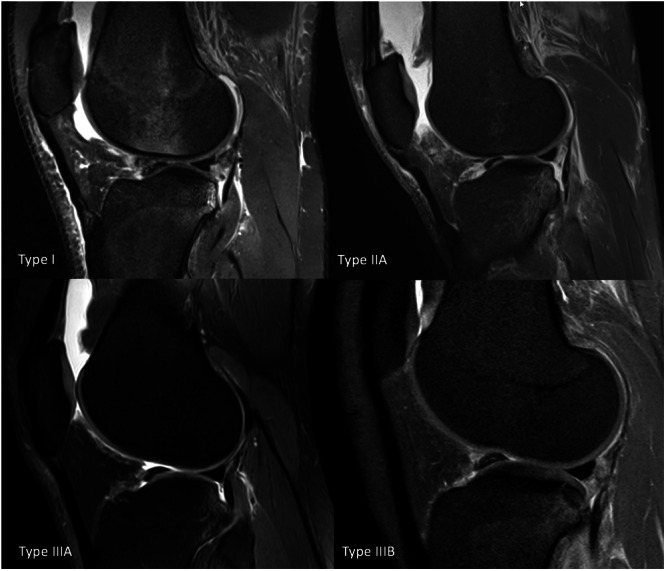
Representation of posterolateral tibial plateau impaction fracture types (according to Bernholt et al. [2]) in the study population examined, in sagittal T2‐weighted magnetic resonance imaging (MRI) scans.

According to the classification system, Type I fractures are defined as posterior buckles of the proximal posterior cortex of the lateral tibial plateau, characterised by a superior to inferior deformity with no involvement of the articular surface. Type II fractures are defined as posterior impaction fractures with involvement of the articular surface, resulting in a decreased lateral tibial plateau depth. They are further differentiated into two subcategories, based on the amount of bone loss present: Type IIA fractures have less than 10% tibial plateau depth bone loss, while Type IIB have greater than 10%. Type III fractures are defined as impaction fractures resulting in a displaced bony fragment. These fractures are also further differentiated into two subcategories: shear fragments (IIIA) and depressed fragments (IIIB) [2] (Figure 2).

### Statistical analysis

Descriptive statistics were performed to determine the incidence of each type of posterolateral tibial plateau impaction fracture, as well as concomitant injuries such as tibial and femoral oedema, meniscal tears, meniscal posterior root tears and medial meniscal ramp lesions.

Patient demographics are presented in mean values and standard deviations. Statistical analyses were carried out using IBM SPSS Statistics Version 27.0.0.0 (IBM Corp., Armonk, New York).

## RESULTS

A total of 556 patients met the inclusion criteria. The mean age of the included study population was 27.8 ± 10.5 years (range, 11–69 years). A total of 164 (29.5%) study participants were females and 392 (70.5%) were males (Table 1).

**Table 1:** Demographic data for patients undergoing primary ACL reconstruction, with and without different types of posterolateral tibial plateau impaction fractures.

			Age (y)	
	*n* (%)	Mean ± SD	Min	Max
No fracture	385 (69.2)	28.0 ± 10.8	11	69
Type I	127 (22.8)	25.5 ± 7.8	14	57
Type IIA	32 (5.8)	32.3 ± 12.1	15	54
Type IIB	0 (0)	–	–	–
Type IIIA	1 (0.2)	37	37	37
Type IIIB	11 (2.0)	35 ± 15.2	19	60
Total	556 (100)	27.8 ± 10.5	11	69

*Note*: Values in parenthesis represent the percentage.

Abbreviations: ACL, anterior cruciate ligament; Max, maximum; Min, minimum; n, number; SD, standard deviation; y, years.

### Incidence of posterolateral tibial plateau impaction fractures

Of the 556 knees with primary ACL reconstruction, 171 (30.8%) posterolateral tibial plateau impaction fractures were identified. 385 (69.2%) patients showed no fracture. The distribution of fracture patterns in the study collective was as follows: Type I: 127 patients; Type IIA: 32 patients; Type IIB: 0 patients; Type IIIA: 1 patient; Type IIIB: 11 patients (Table 1).

### Incidence of concomitant injuries

Of the 556 knees with primary ACL reconstruction, 52.7% showed tibial oedema on T1/T2‐MRI imaging. Femoral oedema was found in 38.5% of cases. Intraoperative arthroscopic examination showed lateral meniscus (LM) tears in 25.9% and medial meniscus (MM) tears in 29.3% of the study population. LM posterior root tears were found in 3.8%, MM posterior root tears in 1.4% and ramp lesions in 7.0% (Table 2).

**Table 2:** Occurrence of different concomitant injuries in patients undergoing primary ACL reconstruction, with and without different types of posterolateral tibial plateau impaction fractures, with a percentage representation of the frequency in relation to the overall occurrence of the respective pathology.

	ACL lesion	Tibial oedema	Femoral oedema	Lateral meniscal tear	Lateral meniscus posterior root tear	Medial meniscal tear	Medial meniscus posterior root tear	Medial meniscal ramp lesion
No fracture	385 (69.2)	123 (42.0)	72 (33.6)	89 (61.8)	14 (66.7)	126 (77.3)	6 (75.0)	29 (74.4)
Type I	127 (22.8)	127 (43.3)	111 (51.9)	46 (31.9)	5 (23.8)	25 (15.3)	0	6 (15.4)
Type IIA	32 (5.8)	31 (10.6)	26 (12.1)	4 (2.8)	1 (4.8)	8 (4.9)	0	4 (10.3)
Type IIB	0 (0)	–	–	–	–	–	–	–
Type IIIA	1 (0.2)	1 (0.3)	0 (0)	0 (0)	0 (0)	0 (0)	0 (0)	0 (0)
Type IIIB	11 (2.0)	11 (3.8)	5 (2.3)	5 (3.5)	1 (4.8)	4 (2.5)	2 (25.0)	0 (0)
Total	556 (100)	293 (100)	214 (100)	144 (100)	21 (100)	163 (100)	8 (100)	39 (100)

*Note*: Values in parenthesis represent the percentage.

Abbreviation: ACL, anterior cruciate ligament.

A 32.2% of the patients with posterolateral tibial plateau fractures suffered an LM lesion, whereas 23.1% of patients without fracture signs showed a LM tear. This proportion was highest in patients with IIIB fractures of the posterolateral tibial plateau. Here, LM tears were found in 45.5% of the patients.

In 29.3% of the patients included in this study, MM tears were found. 21.6% of the patients with evidence of a fracture showed an MM tear, whereas MM lesions were found in 32.7% of the patients without a tibial plateau fracture, using an arthroscopic evaluation.

There were no relevant differences in the percentage of root tears and ramp lesions comparing the fracture and non‐fracture group. A detailed analysis of the diagnosed concomitant injuries in patients with evidence of posterolateral tibial plateau fractures is shown in Table 3.

**Table 3:** Occurrence of different concomitant injuries in patients undergoing primary ACL reconstruction, with and without different types of posterolateral tibial plateau impaction fractures, with a percentage representation of the frequency in relation to overall occurrence of the respective fracture type.

	ACL lesion	Tibial oedema	Femoral oedema	Lateral meniscal tear	Lateral meniscus posterior root tear	Medial meniscal tear	Medial meniscus posterior root tear	Medial meniscal ramp lesion
No fracture	385 (100)	123 (31.9)	72 (18.7)	89 (23.1)	14 (3.6)	126 (32.7)	6 (1.6)	29 (7.5)
Type I	127 (100)	127 (100)	111 (87.4)	46 (36.2)	5 (3.9)	25 (19.7)	0	6 (4.7)
Type IIA	32 (100)	31 (96.9)	26 (81.3)	4 (12.5)	1 (3.1)	8 (25.0)	0	4 (12.5)
Type IIB	0 (0)	–	–	–	–	–	–	–
Type IIIA	1 (100)	1 (100)	0 (0)	0 (0)	0 (0)	0 (0)	0 (0)	0 (0)
Type IIIB	11 (100)	11 (100)	5 (45.5)	5 (45.5)	1 (9.1)	4 (36.4)	2 (18.2)	0 (0)
Total	556 (100)	293 (52.7)	214 (38.5)	144 (25.9)	21 (3.8)	163 (29.3)	8 (1.4)	39 (7.0)

*Note*: Values in parenthesis represent the percentage.

Abbreviation: ACL, anterior cruciate ligament.

## DISCUSSION

The most important findings of this study were that 30.8% of the patients undergoing primary ACL reconstruction showed posterolateral tibial plateau impression fractures, and that LM tears were more frequent in patients with posterolateral tibial plateau impression fractures (with the highest rate of incidence in IIIB fractures). Further important findings were that MM tears are more frequent in patients without posterolateral impaction fractures and that LM posterior root tears are more frequent than MM posterior root tears among the whole study population.

ACL lesions are commonly accompanied by bone bruises and impaction fractures of the posterolateral tibial plateau [6, 9, 10, 11, 12, 14, 16, 17, 18, 19, 20, 27, 29]. The pathomechanism of ACL tears is causal for this, something which can typically be described as a ventral subluxation, valgus and internal rotation mechanism of the tibia. This leads to a high impact of the lateral femoral condyle onto the posterolateral tibial plateau [4, 5, 22, 23, 24]. However, detailed studies dealing with the frequency of posterolateral tibial plateau fractures in patients with ACL lesions are rare. One investigation which deals with this topic was published by Bernholt et al. [1]. The authors investigated 825 patients with ACL lesions, with postulated incidences of 76.8% for lateral tibial plateau bone bruising and 49.3% posterolateral tibial plateau impaction fractures [1].

In the present study, 556 knees with primary ACL reconstruction were investigated. A tibial oedema was detectable in 52.7% of the knee joints examined. At 99.4%, the frequency of tibial oedema was distinctly higher for the group of patients with posterolateral tibial fractures than for patients without fracture evidence (31.9%). The results of this study show lower incidences in patients with ACL lesions in relation for both the occurrence of posterolateral tibial oedema and posterolateral tibial impression fractures. Theoretically, this can be related to a low interobserver reliability in the assessment of posterolateral oedema and impression fractures, though different time intervals between injury and MR imaging may also have caused discrepancies here.

In addition to the incidence of posterolateral tibial plateau fractures, this investigation also focused on concomitant meniscal injuries in patients undergoing primary ACL reconstruction. Intraoperative arthroscopic examination showed LM tears in 144 cases and MM tears in 163 cases. The distribution of the respective MM and LM lesions between patients suffering from posterolateral impression fractures of the tibial plateau and those without tibial fracture is of key interest here. Overall, LM tears were found in 25.9% of the study population. However, 32.2% of the patients suffered an LM lesion, while 23.1% of patients without fracture signs showed an LM tear. This proportion was highest in patients with IIIB fractures of the posterolateral tibial plateau. Here, LM tears were found in 45.5% of the patients. These results coincide with the limited observations of the relevant scientific studies that have been published. Bernholt et al. postulated that the chance of patients with type IIIA and IIIB fractures of the lateral tibial plateau having a complete LM tear was over 3 times higher than for those without type IIIA and IIIB impaction fractures [1].

With an overall frequency of 29.3%, however, MM tears were found more frequently in patients without evidence of fractures (32.7%) than knees with evidence of posterolateral tibial plateau impaction fractures (21.6%). Knowledge about the frequency of accompanying injuries in patients with ACL lesions and their occurrence as a function of different injury patterns is of crucial importance for their detection and the correct choice of therapy.

This is also true for osseous trauma consequences, which is why recognition of the contribution of the osseous geometry of the lateral tibial plateau to knee stability is growing [1]. Biomechanical analysis shows that high‐grade impression fractures of the posterolateral aspect of the tibial plateau increase the instability of ACL‐deficient knees and result in an increase in translational and anterolateral rotational instability [17]. Menzdorf et al. have discussed in this context of posterolateral fractures, where the lateral meniscus loses contact with the tibial plateau. This might have a similar effect to an increased posterior slope [16]. Due to the well‐established relationship between an increased posterior slope and an intensified pivot‐shift, an increased risk of ACL graft failure in patients with displaced posterolateral tibial plateau impaction fractures must be assumed [13, 15, 16]. With regard to the long term results after ACL reconstruction, the distinction between operative and conservative treatment of posterolateral fractures is a key concern. Here, software‐supported simulations of ACL graft loadings depending on the extent of tibial plateau fractures could be a promising future approach.

This study is not without limitations. First, it was conducted using MRI. The use of CT scans could have afforded better detail of the fracture morphologic features, whereby this and comparable studies have shown that posterolateral impression fractures can be detected with sufficient accuracy in MRI. Another limitation is that only descriptive statistics were presented. Due to the study population and the distribution of the different fracture morphologies, comparative analyses were not feasible. Additionally, no patients with concomitant ligamentous injuries of the knee were included, which could potentially lead to a bias in the analysis of posterolateral fractures in patients with ACL lesions.

## CONCLUSIONS

30.8% of the patients who underwent primary ACL reconstruction showed posterolateral tibial plateau impression fractures. LM tears were more frequent in patients with posterolateral tibial plateau impression fractures, with the highest rate of incidence among IIIB fractures. MM tears were more frequent in patients without posterolateral impaction fractures, and LM posterior root tears were more frequent than MM posterior root tears among the whole study population.

## AUTHOR CONTRIBUTIONS

Andreas Fuchs and Kaywan Izadpanah designed the study, collected data, performed the statistical analysis and wrote the manuscript. Andreas Fuchs drafted the manuscript. Andreas Fuchs and Spartak Ovsepyan assessed the MRI scans for posterolateral tibial plateau impaction fractures and concomitant injuries. Spartak Ovsepyan reviewed patients records to determine exact injury patterns and details about concomitant injuries. Andreas Fuchs, RH, Thomas Stein and Matthias Krause helped to design the study, assisted with data interpretation and critically reviewed the manuscript. Tayfun Yilmaz, Thomas Stein and Hagen Schmal helped with data interpretation and critically reviewed the manuscript. All the authors read and approved the final manuscript.

## CONFLICTS OF INTEREST STATEMENT

The authors declare no conflicts of interest.

## ETHICS STATEMENT

The study was approved by the institutional review board of the University Hospital Freiburg (Nr. 91/19 – 210696), and the study was performed in accordance with the Declaration of Helsinki.

## Data Availability

All relevant data is provided within the manuscript. The datasets used and/or analysed during the current study are available from the corresponding author on reasonable request.
